# Nonvolatile phase-programmable spintronic terahertz emitter via laser-induced spin polarization switching

**DOI:** 10.1093/nsr/nwag289

**Published:** 2026-05-18

**Authors:** Shaojie Liu, Zejun Ren, Zehao Yang, Peng Chen, Jiahui Li, Mingcong Dai, Mingxuan Zhang, Deyin Kong, Qiaomei Liu, Lin Bai, Jingdi Zhang, Caihua Wan, Xiaojun Wu

**Affiliations:** International Terahertz Research Center, Hangzhou International Innovation Institute, Beihang University, Hangzhou 311115, China; Department of Physics, Hong Kong University of Science and Technology, Hong Kong 999077, China; School of Electronic and Information Engineering, Beihang University, Beijing 100191, China; School of Electronic and Information Engineering, Beihang University, Beijing 100191, China; Zhangjiang Laboratory, Shanghai 201204, China; Beijing National Laboratory for Condensed Matter Physics, Institute of Physics, Chinese Academy of Sciences, Beijing 100190, China; Center of Materials Science and Optoelectronics Engineering, University of Chinese Academy of Sciences, Beijing 100049, China; Beijing National Laboratory for Condensed Matter Physics, Institute of Physics, Chinese Academy of Sciences, Beijing 100190, China; Center of Materials Science and Optoelectronics Engineering, University of Chinese Academy of Sciences, Beijing 100049, China; School of Electronic and Information Engineering, Beihang University, Beijing 100191, China; School of Electronic and Information Engineering, Beihang University, Beijing 100191, China; School of Electronic and Information Engineering, Beihang University, Beijing 100191, China; Zhangjiang Laboratory, Shanghai 201204, China; International Terahertz Research Center, Hangzhou International Innovation Institute, Beihang University, Hangzhou 311115, China; School of Cyber Science and Technology, Beihang University, Beijing 100191, China; Department of Physics, Hong Kong University of Science and Technology, Hong Kong 999077, China; Beijing National Laboratory for Condensed Matter Physics, Institute of Physics, Chinese Academy of Sciences, Beijing 100190, China; International Terahertz Research Center, Hangzhou International Innovation Institute, Beihang University, Hangzhou 311115, China; School of Electronic and Information Engineering, Beihang University, Beijing 100191, China; Zhangjiang Laboratory, Shanghai 201204, China; Wuhan National Laboratory for Optoelectronics, Huazhong University of Science and Technology, Wuhan 430074, China

**Keywords:** spintronic terahertz emitter, terahertz radiation, nonvolatile terahertz phase encoding, femtosecond laser, antiferromagnetic/ferromagnetic heterostructure

## Abstract

Ultrafast-laser-driven spintronic terahertz (THz) emitters are promising building blocks for future THz technologies, owing to their ability to generate efficient ultrabroadband THz radiation from nanometer-thick metallic heterostructures and to support a variety of functional THz devices. Here, we further extend their functionality by realizing nonvolatile phase encoding in an IrMn_3_/Co_20_Fe_60_B_20_/W heterostructure. By implementing fluence-controlled femtosecond laser excitation, we demonstrate robust THz phase reversal governed by a well-defined threshold of 0.78 mJ/cm², attributed to spin-polarization reversal mediated by exchange bias and magnetic anisotropy manipulation. Time-resolved double-pump experiments show that the THz phase switching is driven by ultrafast laser-induced heating and reveal a thermal gating window of about 15 ps. We further achieve reversible optical writing and magnetic reset between two nonvolatile THz phase states, maintaining a phase contrast above 140% over 30 cycles. Finally, we demonstrate optical-THz spatial phase patterning with a signal-to-noise ratio of 53 dB and a phase contrast of 160%. This work paves the way for write–read–reset THz pattern and information encoding, and advances the integration of STEs with on-chip photonic architectures.

## Teaser

Microscopic bistability in spintronic THz emitters enables laser-induced nonvolatile THz phase reversal and magnetic reset, opening a scalable route toward programmable THz sources and coded THz optics.

## INTRODUCTION

Terahertz (THz) radiation, typically defined in the 0.1–10 THz range, holds great promise for imaging, spectroscopy, high-speed wireless communication, and information processing [1–4]. In recent years, ultrafast-laser-driven spintronic THz emitters (STEs) based on ferromagnet/non-magnet (FM/NM) heterostructures have emerged as one of the leading THz source technologies, owing to their ultrabroadband spectra, high efficiency, nanometer-scale thickness, simple, and low-cost fabrication [5–12]. Their performance is now comparable to that of conventional photoconductive antennas and LiNbO₃-based THz sources [13–15], while offering excellent integrability with planar devices.

On this basis, a variety of spintronic THz functional devices have been demonstrated, including near-field THz microscopy based on STEs [16,17], magnetic-assisted THz manipulation [18,19], fiber-tip STEs [20], ultrabroadband THz detection [21,22], and hybrid STE–metasurface platforms enabling high-speed THz modulation [23], polarization and chirality control [24–26] and beam shaping [27,28]. These spintronic-THz-based functional devices have substantially advanced the state of THz technology and position STEs as a leading platform for future THz systems. However, nonvolatile and rewritable pattern and information encoding implemented directly at the level of the STE remains largely unexplored.

Here, we address this gap by developing a nonvolatile phase-encoded STE based on an IrMn₃/Co₂₀Fe₆₀B₂₀/W heterostructure, capable of robust THz phase reversal under high-fluence femtosecond-laser excitation, which we attribute to laser-induced spin-polarization reversal. The THz phase reversal strongly correlates with the laser fluence and exhibits a pronounced threshold effect at 0.78 mJ/cm². In addition, we demonstrate that THz phase switching is driven by ultrafast laser-induced heating and reveal a thermal gating window of about 15 ps. Furthermore, we realize reversible cycling between two nonvolatile THz phase states by combining optical writing and magnetic reset, and demonstrate spatial THz phase patterning with high signal-to-noise ratio and phase contrast. This work establishes STEs as a platform for nonvolatile, phase-programmable THz pattern and information encoding and opens routes toward programmable THz sources and coded THz optics.

## RESULTS AND DISCUSSION

### Laser-induced spin polarization switching

**Figure 1.**
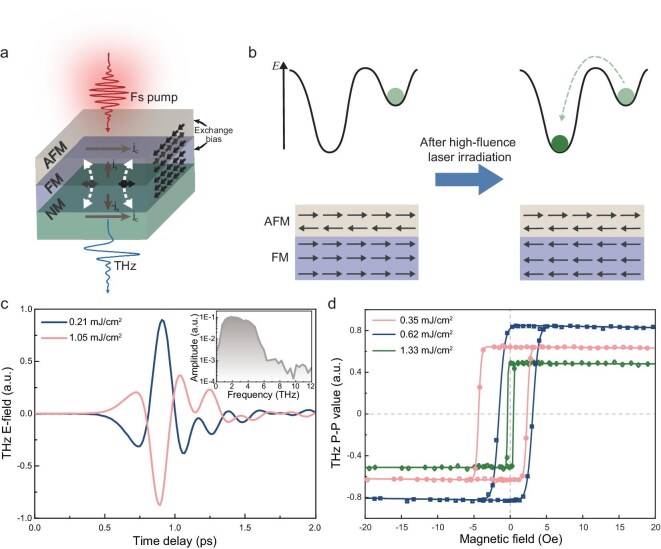
Laser-induced switching of THz phase. (a) Schematic of THz emission from an IrMn_3_/Co_20_Fe_60_B_20_/W heterostructure. In the Co_20_Fe_60_B_20_ layer, the in-plane spin polarization direction is determined by magnetic anisotropy and exchange bias supplied by IrMn_3_. Ultrafast spin currents can be efficiently generated in the FM material through femtosecond laser excitation. These spin currents convert to charge currents in the NM and AFM layers via the ISHE, leading to THz emission. (b) Schematic of FM relevant total energy and AFM/FM heterostructure magnetic properties before and after high laser fluence irradiation. (c) Time-domain waveforms of THz pulses emitted at pump fluence of 0.21 and 1.05 mJ/cm^2^. The inset shows the Fourier-transform spectra. (d) Magnetic field dependence of the THz Peak-to-Peak value measured at pump fluence of 0.35, 0.62, 1.33 mJ/cm^2^, respectively.

Figure 1a illustrates the fundamental principle of a nonvolatile phase-programmable STE based on a trilayer structure comprising antiferromagnet (AFM), FM, and NM metal thin films. The AFM/FM/NM trilayer heterostructure, with a stacking sequence of IrMn_3_(2 nm)/Co_20_Fe_60_B_20_ (2 nm)/W (2 nm), was fabricated by magnetron sputtering at a base chamber pressure below 2 × 10^−8^ Torr. An in-plane external magnetic field of 180 Oe was applied to induce the exchange bias between AFM and FM layers and establish the magnetic anisotropy of the FM layer. The NM layer enhances spin-charge conversion and amplifies the THz transient intensity [9]. An incident femtosecond laser pulse, with a central wavelength of 800 nm, a pulse duration of 35 fs, a repetition rate of 1 kHz, and a 10 mm beam diameter (1/e^2^), excites electrons in the FM layer to states above the Fermi energy, thereby altering their band velocity and scattering rate. This excitation generates a longitudinal spin-polarized current (${j}_s$), which comprises both upward- and downward-flowing components due to the different densities and mobilities of spin-up and spin-down electrons [9,10,29]. Upon entering the AFM and NM layers, the spin-orbit field causes spin-up and spin-down electrons to deflect in opposite directions, converting the spin currents into a transverse in-plane charge current (${j}_c$) via the inverse spin Hall effect (ISHE) [6]. Due to the opposite spin Hall angles $\gamma $ of IrMn_3_ and W, these charge currents flow in the same direction. This ultrafast charge current, hence, results in THz emission.

The radiation mechanism can be described by ${E}_{THz} \propto {j}_c \propto ( {{\gamma }_{IrM{n}_3} \cdot {j}_{s - IrM{n}_3} + {\gamma }_W \cdot {j}_{s - W}} ) \times {\mathrm{{\bf{M}}}}/| {\mathrm{{\bf{M}}}} |$, where ${\mathrm{{\bf{M}}}}$ denotes the magnetization vector (spin polarization direction) of the FM layer. THz transient phase direction is determined by the magnetization vector, which, when manipulated, is expected to enable ultrafast information storage and retrieval. To achieve this, the FM layer is engineered to support two magnetization states, which can be manipulated by femtosecond laser and magnetic field.

The magnetization vector direction of the FM layer is determined by two types of energy. The first is the exchange bias energy, described by ${E}_e = - \textit{J cos}\theta $, where *J* represents the interface exchange energy coefficient. The second is the magnetic anisotropy energy of the FM layer, induced by the in-plane magnetic field applied during fabrication, this energy is given by ${E}_m = - K{\boldsymbol M}_sco{s}^2\theta $, where *K* is the induced anisotropic constant, ${\boldsymbol M}_s$ is the direction of the FM magnetic moment which can be ± 1, and $\theta $ is the angle between the exchange bias field direction and FM magnetic moment direction. The total energy of the FM layer, given by $E = {E}_e + {E}_m = - \textit{J cos}\theta - K{M}_sco{s}^2\theta $, determines the two states based on the sign of ${\boldsymbol M}_s$. When ${\boldsymbol M}_s$ opposes the exchange bias direction, the FM system is in a metastable state, with spin polarization in the ‘right’ direction. Conversely, when ${\boldsymbol M}_s$ aligns with the exchange bias direction, the FM system is in a more stable state, with spin polarization in the ‘left’ direction, as shown in Fig. 1b.

The blue line of Fig. 1c presents the high signal-to-noise ratio THz temporal waveform generated by the IrMn_3_/Co_20_Fe_60_B_20_/W heterostructure using laser irradiation at the low fluence (0.21 mJ/cm²). The corresponding spectrum, exhibiting a spectral width of up to 6 THz, is provided in the inset. Subsequently, high-fluence laser pulse transfers the FM system from the metastable state to the stable state, thereby switching the spin polarization direction, as illustrated in Fig. 1b. The phase-reversed THz temporal waveform recorded under laser irradiation with a fluence of 1.05 mJ/cm² is shown by the pink line in Fig. 1c and exhibits a shorter pulse duration (see Note S2 for more details). By alternating between low and high pump laser fluences, the THz phase was inverted, attaining a contrast ratio of nearly 200%. This high signal-to-noise ratio nonvolatile phase reversal opens up a new path for optical-THz pattern and information encoding.

Figure 1d records the THz peak-to-peak value as a function of the applied magnetic field. The THz hysteresis (THz-H) behavior under low fluence pump conditions is evident and comparable to the magnetic hysteresis loop observed via magneto-optical measurements [6,30]. The exchange bias (*H*_EB_) and coercivity (*H*_c_) are calculated to be −1 Oe and 3.35 Oe, respectively, under a pump fluence of 0.35 mJ/cm². As the pump laser fluence ascends, the laser-induced transient effective temperature at the interface gradually rises (especially at the center of the beam), eventually surpassing the blocking temperature of the AFM [31,32]. This results in the softened coupling of the exchange bias effect at the FM/AFM interface as well as the reduction in *H*_c_. The THz peak values initially increase and then diminish, suggesting that before decoupling, the rise in pump fluence enhances spin current generation. Post-decoupling, the conversion efficiency from spin currents to charge currents diminishes, resulting in a reduction of THz wave amplitude.

### Threshold behavior of THz phase reversal

**Figure 2.**
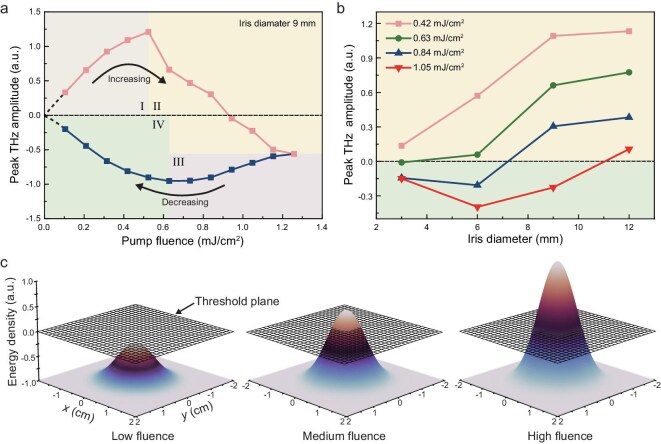
Dependence of THz phase reversal on the pump laser fluence and beam size. (a) Evolution of peak THz amplitude with pump fluence at a 9 mm diameter laser beam. (b) Peak THz amplitude as a function of the iris diameter under various pump fluences. (c) Schematic showing the relationship of Gaussian-profile laser fluence on the THz phase reversal threshold plane.

To investigate the impact of pump fluence on THz phase reversal, Fig. 2a shows the alterations in the peak THz amplitude as the pump fluence increases and decreases, maintaining a laser diameter of 9 mm by iris. This can be divided into four stages: I, II, III, and IV. In stage I, the peak THz amplitude exhibits an increasing trend with an applied fluence of up to 0.52 mJ/cm², consistent with previous reports [6]. In stage II, the peak THz amplitude continues to diminish as the increasing pump fluence up to 1.25 mJ/cm². A complete phase reversal of the emitted THz pulse occurs at a fluence above 0.93 mJ/cm², and further increasing the pump fluence up to 1.75 mJ/cm² leads to sample damage, resulting in a decrease in the peak THz amplitude (see Fig. S3). In stage III, following the phase reversal, the peak THz amplitude increases as the pump fluence decreases from its maximum amplitude, influenced by the spin current magnitude and the exchange bias coupling efficiency. At this stage, high-fluence pumping partially decouples the exchange bias, which reduces the efficiency of converting spin current into charge current and thus suppresses the THz emission. However, as the pump fluence decreases, the spin-current yield does not drop significantly. This causes the peak THz intensity increases again. In stage IV, further reduction in pump fluence leads to a decrease in the peak THz amplitude, which does not return to its original phase, primarily due to the reduction in the yield of spin current.

Furthermore, the relationship between the peak THz amplitude and the iris-controlled pump-spot diameter under various pump fluences is presented in Fig. 2b (see Fig. S4). At a pump fluence of 0.42 mJ/cm², the peak THz amplitude remains positive and increases as the iris diameter enlarges. The THz phase reversal occurs for various iris diameters as the laser fluence rises. For pump fluences of 0.84 mJ/cm² and 1.05 mJ/cm², the THz phase and peak amplitude remain consistent within a 3 mm iris diameter, indicating that the spin polarization direction of the FM has completely reversed under the pump fluence above 0.84 mJ/cm². As the iris expands, the THz phase shifts from negative to positive, suggesting that the pump fluence at the edge of the laser spot is insufficient to achieve THz phase reversal.

To elucidate this phenomenon, a 3D illustration displayed in Fig. 2c delineates the spatial relationship between Gaussian-profile laser pulses and the THz phase reversal threshold plane under three different pump fluences. As the laser fluence rises, THz phase reversal is initially witnessed in the central region of the laser spot and subsequently expands outward. Ultimately, THz waves are documented by the detector through coherent superposition.

### Ultrafast thermal gating in double-pump THz phase switching

**Figure 3.**
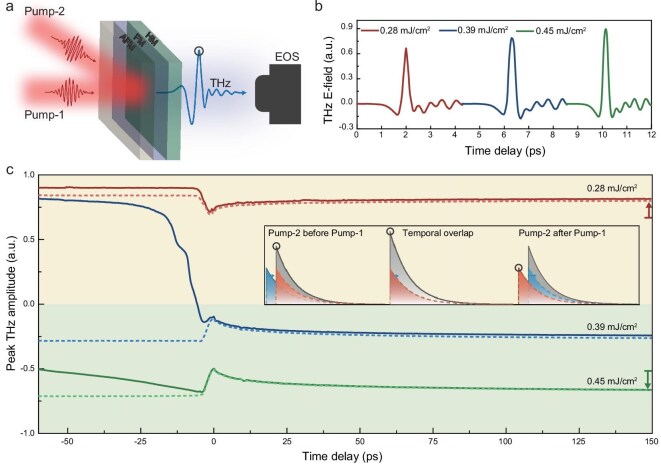
THz phase switching under double-pump excitation. (a) Schematic of the double-pump THz detection system. The fluences of Pump-1 and Pump-2 are equal, and the THz transients and peak amplitudes are detected by electro-optic sampling (EOS). (b) THz transients generated by excitation with Pump-1 at three different fluences. (c) Temporal dynamics of the peak THz amplitude induced by the double-pump beams at three different fluences. The solid and dashed lines represent the first and second PTTRMs, respectively. The inset illustrates the relationship between the double-pump pulse delay and the laser-induced ultrafast thermal effect using the two-temperature model. The red and green lines are vertically offset upwards and downwards, respectively, for clarity.

To clarify the temporal conditions under which the laser-induced THz phase switching is triggered, we performed a double-pump THz detection experiment as shown in Fig. 3a. Pump-2, matched in fluence to Pump-1, was incident at 30° with a controllable delay, while the THz emission generated by Pump-1 excitation was detected by electro-optic sampling. Figure 3b presents the initial THz transients of the sample at Pump-1 fluences of 0.28, 0.39, and 0.45 mJ/cm^2^.

Figure 3c shows how the ultrafast thermal effect generated by a delay-tunable double pump pulse modulates the peak THz amplitude at these three excitation fluences. The horizontal axis gives the pump–pump delay: Pump-2 approaches Pump-1 from negative delay, coincides at zero delay, and then recedes at positive delay. At a double-pump fluence of 0.28 mJ/cm², the first Photo-Thermal Time-Resolved Measurement (PTTRM; red solid line) is nearly constant for delays earlier than −7 ps, decreases from −7 ps to 0 ps as ultrafast thermal superposition slightly perturbs the spin polarization near the sample center, and then gradually stabilizes within a few hundred picoseconds after temporal overlap. The second PTTRM (red dotted line) closely reproduces the first, with a marginal decrease and no phase switching, indicating that this fluence is insufficient to alter the spin polarization across the pumped area.

At the double-pump fluence of 0.39 mJ/cm² (blue solid line), the THz peak initially decreases slowly and then collapses in an avalanche-like manner as Pump-2 approaches Pump-1, culminating in a phase reversal, after which the signal stabilizes, indicating that the THz phase reversal is governed by ultrafast thermal control induced by the two pulses and that, under this configuration, a ∼15 ps thermal gating window is realized, corresponding to a magnetization-switching threshold fluence of 0.78 mJ/cm².

The second PTTRM (blue dashed line) differs markedly, showing a negative, stable THz peak, where the laser-induced ultrafast thermal superimposition is insufficient to modify the THz yield, and a reduced absolute peak amplitude from −4.5 ps to 0 ps due to substantial thermal overlap that perturbs the AFM/FM exchange bias effect [30]. Laser-induced ultrafast thermal effects can be qualitatively depicted by the two-temperature (2T) model as illustrated in the inset for three representative double-pump delays (Pump 2 leading, temporal overlap, and Pump 2 lagging). Circles mark the total deposited thermal energy at the detection position, which correlates with the peak THz amplitude. Varying the delay changes the deposited energy and thus directly tunes the THz peak. The double-pump pulses rapidly deposit energy as heat into the metallic stack; electrons and phonons thermalize within sub-picoseconds (see Fig. S6). When the deposited thermal energy exceeds a critical threshold, the transient temperature rise reduces the magnetic anisotropy of the FM layer, so that the magnetization, initially governed by the FM anisotropy, becomes dominated by the exchange-bias energy and aligns along the pinned direction, resulting in a reversal of the THz polarity.

At the double-pump fluence of 0.45 mJ/cm² (green solid line), the first PTTRM shows that the THz phase is already reversed for all delays. As Pump-2 approaches Pump-1 from negative delay, the peak THz amplitude gradually decreases, which we attribute to the increasing contribution of regions where the thermal effects of the two pulses superimpose and drive the near-threshold part of the Gaussian spot further above the switching threshold. A second PTTRM at the same fluence shows an almost delay-independent THz peak, similar to the second PTTRM at 0.39 mJ/cm², indicating that the system has already relaxed into a stable reversed-phase state.

### Reconfigurable nonvolatile THz phase states

Furthermore, the ability to reversibly write and erase the nonvolatile THz phase state greatly enhances the reconfigurability and reuse of the emitter, without requiring any structural modification of the device. A modest external magnetic field provides a straightforward and reliable means to reset the THz phase in regions that have been switched by high-fluence laser excitation.

**Figure 4.**
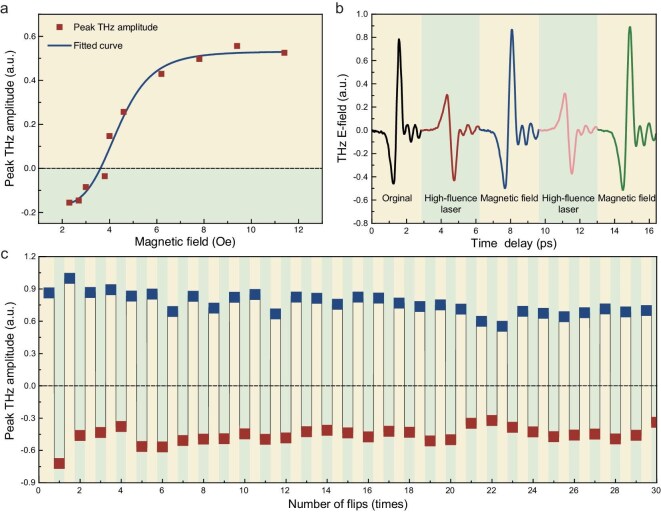
Optical–magnetic cooperative control of the THz phase. (a) Peak THz amplitude under low-fluence excitation as a function of in-plane magnetic field strength after the sample has been driven into the reversed phase state by a high-fluence pulse. (b) THz waveforms measured with a low-fluence read-out pulse while the sample is alternately driven by high-fluence ‘write’ pulses and external magnetic ‘reset’ fields. (c) Peak THz amplitudes for 30 consecutive write-reset cycles at the same location.

To determine the external magnetic field required to reset the THz phase, we measured the peak THz amplitude under low-fluence excitation as a function of in-plane magnetic field, as shown in Fig. 4a. As the field strength increases, the peak amplitude grows, undergoes a phase reversal, and then saturates for fields larger than about 7 Oe, indicating that the magnetization is fully realigned along the field direction. Figure 4b displays the THz waveforms under low-fluence excitation when the sample is alternately subjected to a high-fluence pulse and an external magnetic field. The high-fluence pulse optically writes the reversed THz phase state, whereas the applied magnetic field restores the original phase state, realizing a reversible write–reset operation. Keeping the external field at 8 Oe and the high-fluence pulse at 1.0 mJ/cm², we performed 30 consecutive write–reset cycles at the same location, as illustrated in Fig. 4c. The contrast between adjacent THz peaks consistently exceeds 142%, demonstrating robust, repeatable control of the nonvolatile THz phase state.

### Spatially programmable THz phase pattern

**Figure 5.**
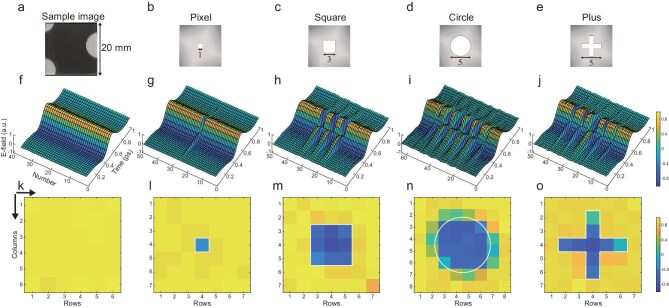
Spatial THz phase patterning. (a) Surface image of the IrMn_3_/Co_20_Fe_60_B_20_/W heterostructure. The sample emits reference THz transients (f) and 2D peak THz amplitudes (k). Patterns for pixel (b), square (c), circle (d), and plus (e) shapes was stored on the sample using high pump laser fluence. The radiated THz transients from the sample were recorded in the middle layer (g, h, i, j) via a 2D scan using a 1 × 1 mm^2^ with low-fluence pump laser fluence. The 2D peak THz amplitudes, which display the stored pattern, are shown in the lower layer (l, m, n, o).

Finally, we demonstrate spatially programmable THz phase patterns in the IrMn_3_/Co_20_Fe_60_B_20_/W heterostructure. Figure 5a shows an optical image of the 6 nm-thick sample. Using a low-fluence pump and a 1 × 1 mm^2^ unit cell, we map the THz emission, acquiring time-domain waveforms (Fig. 5f) and two-dimensional (2D) peak-amplitude maps (Fig. 5k); the response is essentially uniform across the scanned area. A high-fluence pulse is then used to write a pattern defined by a pixel square aperture (Fig. 5b), and a subsequent low-fluence scan reads out the modified state (Fig. 5g), from which the extracted THz peaks yield the corresponding 2D intensity distribution (Fig. 5l). The signal-to-noise ratio reaches 53 dB, and the polarity contrast between positive and negative signals is about 160%, enabling robust binary (0/1) phase encoding. Using the same write–read protocol, square (Fig. 5c), circle (Fig. 5d), and plus (Fig. 5e) patterns are inscribed onto the emitter. Figures 5h–j and 5m–o present the corresponding THz waveforms and 2D peak maps, respectively, obtained under low-fluence read-out. These results show that the nonvolatile phase-programmable STE can serve as a simple platform for writing and reading THz phase patterns by alternating high- and low-fluence excitation.

## CONCLUSION

In summary, the contactless nonvolatile phase-programmable STE we designed features an IrMn_3_/Co_20_Fe_60_B_20_/W heterostructure, enabling nonvolatile THz phase reversal by high-fluence femtosecond laser, with a threshold of 0.78 mJ/cm². Ultrafast laser-induced heating–driven spin-polarization reversal has been demonstrated by double-pump measurements, and the reversed THz phase can be restored by applying an external magnetic field. We constructed a nonvolatile, reconfigurable STE characterized by high-fluence optical writing, low-fluence read-out, and magnetic reset, highlighting its potential as a platform for THz phase and pattern encoding. Our results open routes toward programmable STEs, coded THz optics, and their integration with on-chip photonic architectures.

## METHODS

### Sample fabrication

The double-sided polished quartz substrate was ultrasonically cleaned in ethanol for 30 minutes. Subsequently, it was placed into the vacuum chamber of the sputtering system, and an in-plane magnetic field of 180 Oe was applied. After evacuating the chamber to a base pressure below 10⁻⁸ Pa, a W/Co_20_Fe_60_B_20_/IrMn_3_ multilayer film was sequentially deposited via magnetron sputtering. The thickness of each layer (W, Co_20_Fe_60_B_20_, IrMn_3_) was precisely controlled to 2 nm using calibrated deposition rates and timing. Application of the in-plane magnetic field during deposition induced uniaxial magnetic anisotropy in the FM layer and established an exchange bias between the AFM layer and the FM layer.

### THz emission spectroscopy

THz emission measurements were performed using a femtosecond laser source with a central wavelength of 800 nm, a pulse duration of 35 fs, and a repetition rate of 1 kHz. A tunable-energy pump laser beam directly excited the sample to generate THz radiation. The emitted THz waves were collimated and focused onto a ZnTe/GaP detection crystal using parabolic mirrors. A time-delayed probe laser beam was co-focused with the THz radiation onto the same ZnTe/GaP crystal. THz wave detection was implemented via electro-optic sampling (EOS), utilizing the ZnTe/GaP crystal as the sensor, followed by a quarter-wave plate, a Wollaston prism, and a balanced photodetector to measure the polarization rotation induced by the THz electric field. The magnetic field involved in the experiment is achieved by an electromagnet with controllable magnetic field strength. All measurements were conducted at room temperature.

## Supplementary Material

nwag289_Supplemental_File

## Data Availability

The data that support the findings of this work are available from the corresponding author upon reasonable request.
