# Supplementary material for: Nonvolatile phase-programmable spintronic terahertz emitter via laser-induced spin polarization switching
Source: Natl Sci Rev. 2026 May 18;13(12):nwag289. doi: 10.1093/nsr/nwag289 (PMC13263117; doi:10.1093/nsr/nwag289)
Supplement: nwag289_Supplemental_File [file nwag289_supplemental_file.pdf]

Supplementary Data

Nonvolatile Phase-Programmable Spintronic Terahertz Emitter via Laser-induced Spin Polarization Switching

Shaojie Liu, Zejun Ren, Zehao Yang, Peng Chen, Jiahui Li, Mingcong Dai, Mingxuan Zhang, Deyin Kong, Qiaomei Liu, Lin Bai, Jingdi Zhang, Caihua Wan and Xiaojun Wu*

Contents:

S1. The experiment setup 3

S2. Fourier analysis of the THz waveforms under low- and high-fluence excitation 4

S3. THz peak value as function of pump laser energy 5

S4. The relationship between THz peak value and iris-controlled pump-spot diameter 5

S5. Laser-induced ultrafast demagnetization 6

S6. Two temperature model 7

S7. The physical pictures of pixel, square, circle, and plus shapes 8

S8. THz field strength calculation 8

S1. The experiment setup

The schematic diagram of the experimental setup is shown in Figure S1. A femtosecond laser pulse with a central wavelength of 800 nm, a pulse duration of 40 fs, a repetition rate of 1 kHz incident enters the optical system. This system has three functions.

Optical pump THz detection setup: The incident femtosecond laser is divided into pump 1 and probe beam using a beam splitter. The pump beam excites the sample to generate THz radiation, while the probe beam passes through an optical delay 1 and pumped onto ZnTe/GaP crystal. The THz wave is detected using an Electro-Optic Sampling, which involves components such as ZnTe/Gap crystal, a quarter-wave plate, a Wollaston prism, and a balanced detector.

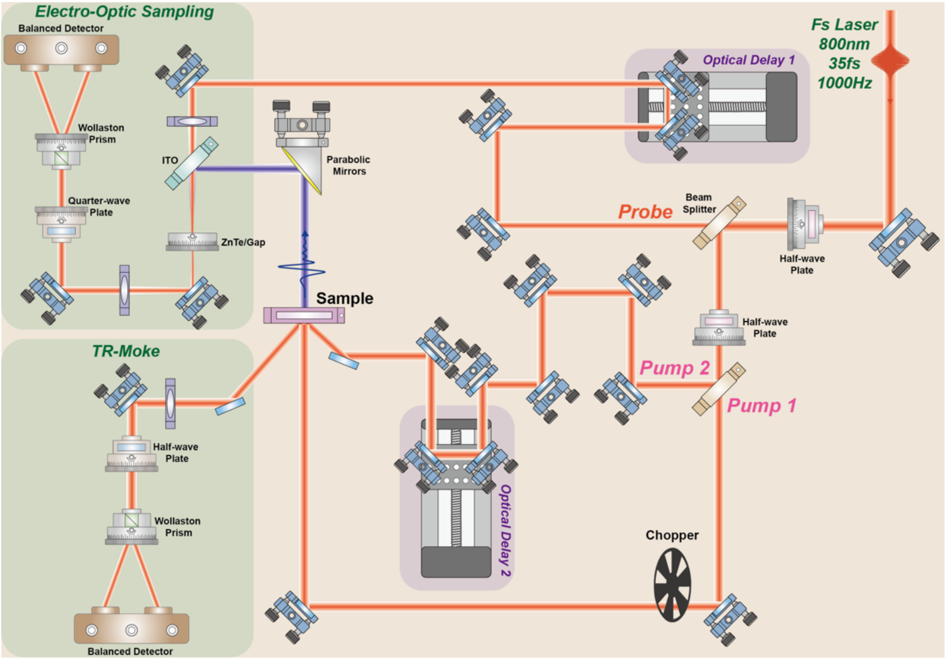

Figure S1. Schematic diagram of the experimental setup.

TR-Moke setup: Two laser beams, an 800 nm pump (Pump 1 in Figure S1) and an 800nm probe (Pump 2 in Figure S1), were used under time-synchronized conditions to illuminate the sample. Pump 1 was directed at the sample at normal incidence, while the probe beam (Pump 2) illuminated the sample at a 15-degree angle. The reflected probe polarization was recorded using time-resolved magneto-optic Kerr effect (TR-MOKE) measurements, which involved a half-wave plate, a Wollaston prism, and a balanced detector, by varying the time delay between the pump and probe lasers.

Double-pump setup: Utilizing the optical pump THz detection setup, we introduced

a second pump beam (Pump 2) with energy equivalent to Pump 1, illuminating the sample at a 15-degree angle. By adjusting the optical delay between Pump 1 and Pump 2, the THz peak value changes were observed.

S2. Fourier analysis of the THz waveforms under low- and high-fluence excitation

To further analyze the apparent difference between the THz waveforms obtained under low- and high-fluence excitation in Fig. 1c, we performed a Fourier transform of the corresponding time-domain signals. The extracted amplitude spectra and spectral phase are presented in Fig. S2.

As shown in Fig. S2, the two spectra exhibit similar overall profiles, but with a noticeable difference in their spectral-weight distribution. Compared with the low-fluence case, the THz signal measured under high-fluence excitation contains relatively reduced low-frequency components and enhanced high-frequency components, indicating a broader spectral bandwidth. By contrast, the spectral phase remains largely unchanged over the measured frequency range.

These results suggest that the main difference between the two THz waveforms is associated with a change in the temporal width of the emitted THz transient. The broader spectrum observed under high-fluence excitation is consistent with a shorter THz pulse duration in the time domain. Therefore, the high-fluence waveform may appear to show a slight shift in the peak position when directly compared with the low-fluence waveform. This indicate that the spin-polarization states before and after phase reversal are not perfectly symmetric in their ultrafast dynamics.

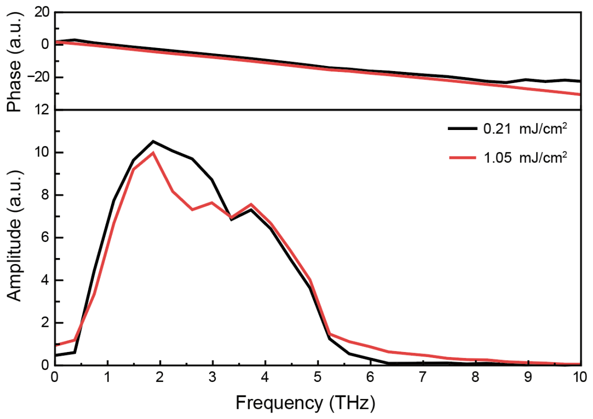

Figure S2. The amplitude spectra and spectral phase compare under the low- and high-fluence excitation.

S3. THz peak value as function of pump laser energy

Figure S3 shows the THz peak value as a function of the pump fluence after 9 mm iris diameter. When the pump light fluence is below 1.75 mJ/cm², the variation in THz peak intensity aligns with the findings presented in the main text. However, when the pump light fluence exceeds 1.75 mJ/cm², the laser damages the emitter, leading to a reduction in THz peak value. This effect becomes more pronounced as the laser fluence increases.

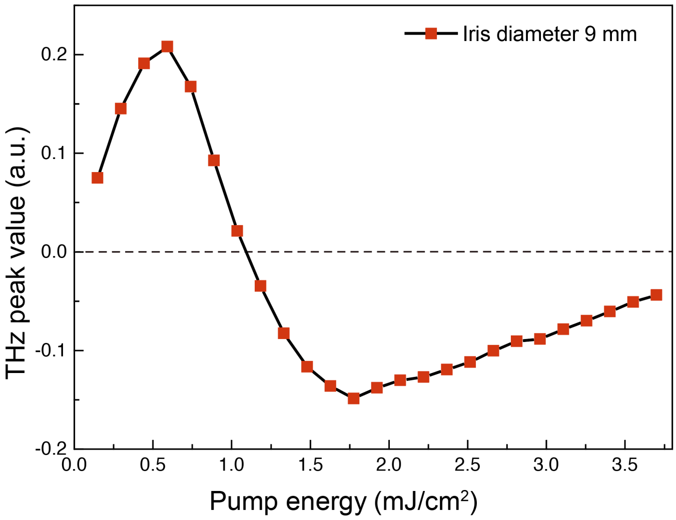

Figure S3. The THz peak value changes with the pump fluence increasing at 9 mm laser diameter.

S4. The relationship between THz peak value and iris-controlled pump-spot diameter

The relationship between the increasing and decreasing of pump laser fluence and the THz peak value under different iris-controlled pump-spot diameters is shown in Figure S4. The extracted relationship between of them is presented in Figure 2B.

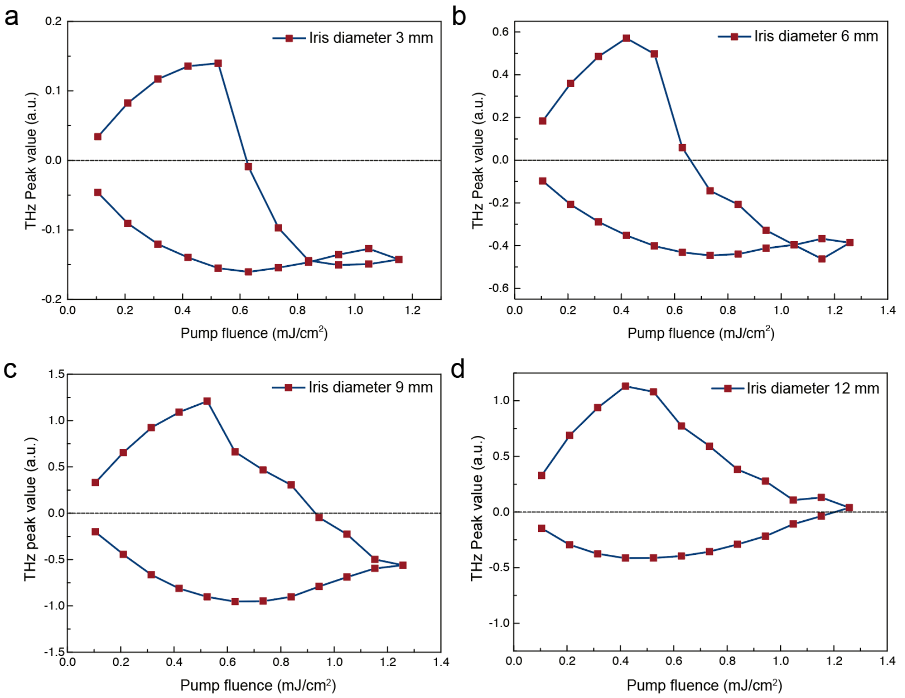

Figure S4. The THz peak value changes with the pump fluence increasing and decreasing at 3mm, 6mm, 9mm, and 12mm iris-controlled laser-spot diameters.

S5. Laser-induced ultrafast demagnetization

Figure S5 illustrates the ultrafast demagnetization of the trilayer heterostructure at different pump fluences in the absence of an external magnetic field, measured by a custom-built time-resolved magneto-optical Kerr effect (TR-MOKE) setup, demonstrating that ultrafast demagnetization can occur within 3 ps.

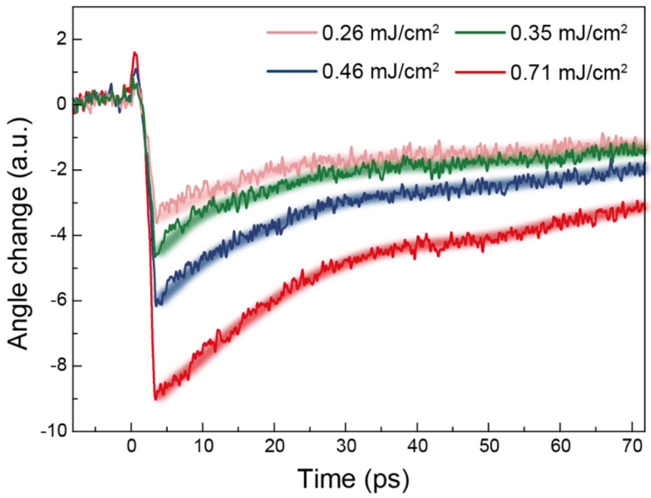

Figure S5. Laser-induced ultrafast demagnetization in $IrMn_3/Co_{20}Fe_{60}B_{20}/W$ heterostructures under various pump fluence.

S6. Two temperature model

The ultrafast thermal dynamics induced by laser excitation in the $IrMn_3/Co_{20}Fe_{60}B_{20}/W$ heterostructure can be effectively described by the Two-Temperature Model (2T Model). Given the negligible total thickness, the material stack can be characterized by a single temperature. The 2T model accounts for the distinct thermal dynamics of electrons and phonons, incorporating effects of electron and lattice temperatures, as well as heat diffusion. The governing equations are expressed as follows:

$$C_e(T_e)\frac{dT_e}{dt} = -G_{el}(T_e - T_l) + S(t) - \frac{C_e}{\tau_{decay}}(T_l - T_R) \tag{S1}$$

$$C_l\frac{dT_l}{dt} = G_{el}(T_e - T_l) \tag{S2}$$

Where T_e and T_l are electron and lattice temperatures, C_e and C_l denote electron and lattice specific heat capacities, with estimated value of $1.8e^5$ J/m^3/K and $2.2e^5$ J/m^3/K, $S(t)$ is the volumetric laser power density, G_{el} is the electron-phonon coupling constant, valued of $5.8e^{17}$ W/m^3/K, τ_{decay} is the heat diffusion time, estimated to be 100 ps, T_R is the room temperature.

When the pump laser intensity is 0.4mJ/cm^2, the estimated temperature changes are shown in Figure S6, showing a rapid increase to a maximum within 0.2 ps when the laser is pumped, followed by a decrease due to heat diffusion. If the induced temperature increase exceeds the threshold for spin polarization switching, it results in a reversal of the THz phase, as observed in the experiment. The 2T dynamics induced by the double-laser pump have been inserted in Figure 3c.

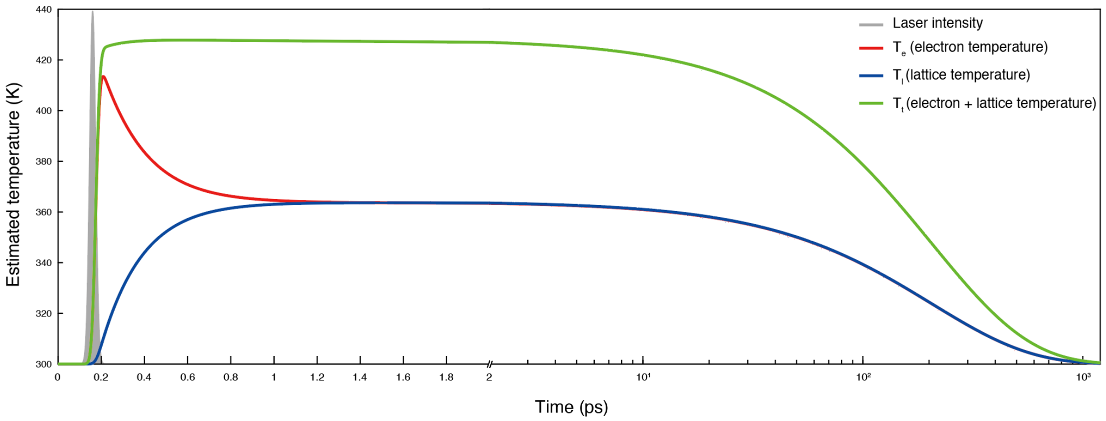

Figure S6. Estimated temperature of $IrMn_3/Co_{20}Fe_{60}B_{20}/W$ heterostructure as a function of time used to calculate the temperature dynamics by 2T model.

S7. The physical pictures of pixel, square, circle, and plus shapes

The physical picture of the metal iron sheet with a pixel aperture, a square aperture, a circle aperture, and a plus aperture is shown in Figure S7. The sizes of these apertures correspond to those depicted in Figures 4B, 4C, 4D, and 4E.

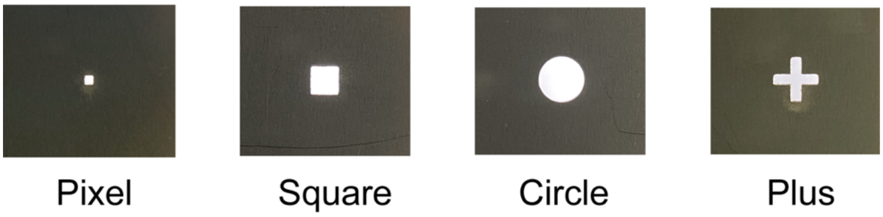

Figure S7. The physical pictures of pixel, square, circle, and plus shapes.

S8. THz field strength calculation

We have now estimated the absolute THz electric-field strength based on the electro-optic sampling (EOS) measurement under the pump laser with a fluence of 0.42 mJ/cm^2, a central wavelength of 800 nm and a pulse duration of 35 fs. The THz field is obtained from the EOS signal according to[1,2]:

$$E_{THz} = \frac{\lambda}{2\pi n_0^3 r_{41} t_{filter} L_{eff}} \arcsin\left(\frac{I_1 - I_2}{I_1 + I_2}\right) \quad \text{(S3)}$$

Where, $\lambda = 800nm$ is the probe wavelength, $n_0 = 3.2$ is the refractive index of the GaP detection crystal at the probe wavelength, $r_{41} = 0.88\, pm/V$ is the electro-optic coefficient, $L_{eff} = 0.2mm$ is the effective thickness of the detection crystal, and $t_{filter} = 0.8$ is the transmission coefficient of the THz low-pass filter. From the balanced detector, the measured modulation depth $(I_1 - I_2)/(I_1 + I_2)$ is approximately 0.05. Using these parameters, we estimate the peak THz electric field to be about 13.8 kV/cm.

[1] ***Applied Physics Letters*** 98.9 (2011).

[2] ***Journal of the Optical Society of America B*** 18.3 (2001): 313-317.
